# First whole-genome sequence of *Triatoma sanguisuga* (Le Conte, 1855), vector of Chagas disease

**DOI:** 10.1093/g3journal/jkae308

**Published:** 2024-12-31

**Authors:** Jennifer K Peterson, Madolyn L MacDonald, Vincenzo A Ellis

**Affiliations:** Department of Entomology and Wildlife Ecology, University of Delaware, Newark, DE 19716, USA; Bioinformatics and Computational Biology Core, University of Delaware, Newark, DE 19716, USA; Department of Entomology and Wildlife Ecology, University of Delaware, Newark, DE 19716, USA

**Keywords:** *Triatoma sanguisuga*, triatomine bugs, *Trypanosoma cruzi*, Chagas disease, disease vectors

## Abstract

*Triatoma sanguisuga* is the most widespread triatomine bug species in the United States. The species vectors the human parasite *Trypanosoma cruzi*, which causes Chagas disease. Vector-borne Chagas disease is rarely diagnosed in the United States, but *T. sanguisuga* has been implicated in a handful of cases. Despite its public health importance, little is known about the genomics or population genetics of *T. sanguisug*a. Here, we used long-read sequencing to assemble the first whole-genome sequence for *T. sanguisuga* using DNA extracted from 1 adult specimen from Delaware. The final size of the genome was 1.162 Gb with 77.7× coverage. The assembly consisted of 183 contigs with an N50 size of 94.97 kb. The Benchmarking Universal Single-Copy Ortholog complete score was 99.1%, suggesting a very complete assembly. Genome-wide GC content was 33.56%, and DNA methylation was 18.84%. The genome consists of 62.75% repetitive DNAs and 17,799 predicted coding genes. The assembled *T. sanguisuga* genome was very close in size and BUSCO score to that of Triatominae species *T. dimidiata* (1.16 Gb with 99.1% BUSCO score for *T. sanguisuga* vs 1.22 Gb with 98.7% BUSCO score for *T. dimidiata*) and slightly larger than that of *T. infestans* and *Rhodnius prolixus* (949 Mb with 90.4% BUSCO score and 706 Mb with 96.5% BUSCO score, respectively). The *T. sanguisuga* genome is the first North American triatomine species genome to be sequenced, allowing for deeper investigations into epidemiologically relevant aspects of triatomines in temperate climates, thus providing potential vector-borne disease management targets and strengthening public health preparedness.

## Introduction

Triatomine bugs (“triatomines”) are hematophagous (i.e. blood-feeding) arthropods that feed on a wide variety of vertebrate host species, including humans. Triatomines are of epidemiological interest due to their harborage of the protozoan parasite *Trypanosoma cruzi*, the causative agent of Chagas disease in humans. If left untreated, infection with *Try. cruzi* is lifelong and can lead to serious cardiac and gastrointestinal alterations over time (Rassi Jr *et al*. 2010). There are 162 described species of triatomine bugs (159 extant and 3 fossil species; Alevi *et al*. 2021; Zhao *et al*. 2021, 2023; Oliveira Correia *et al*. 2022, 2024), of which 11 are found in the United States (Bern and Montgomery 2009; Bern *et al*. 2011, 2019).

**Fig. 1.**
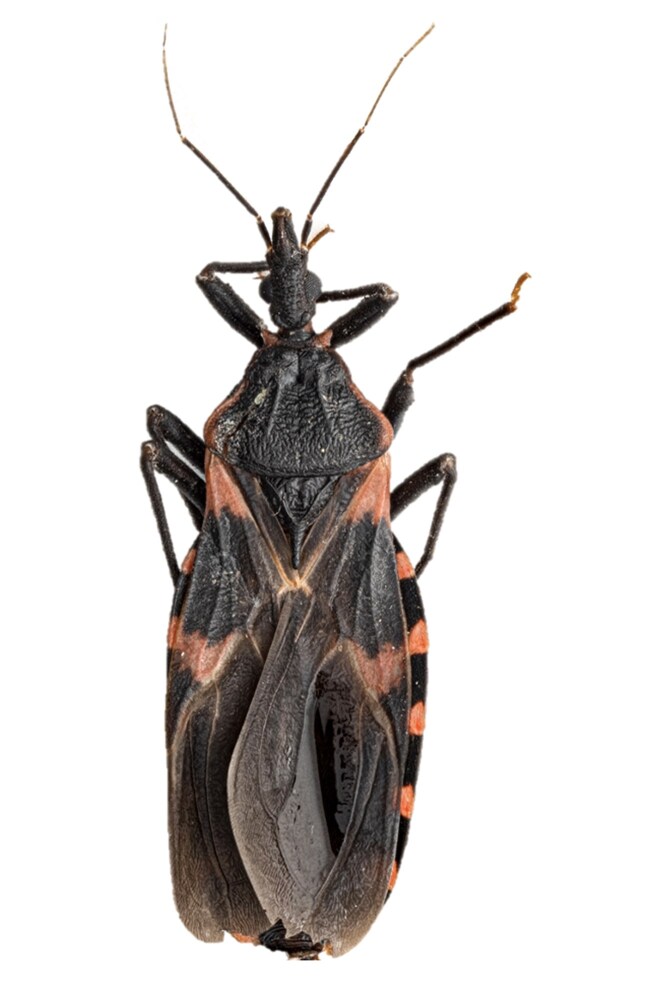
*T. sanguisuga* specimen used in genome sequencing. Image first appeared in Fig. 2b (Peterson *et al*. 2024). Photo taken by Solomon Hendrix.

**Fig. 2.**
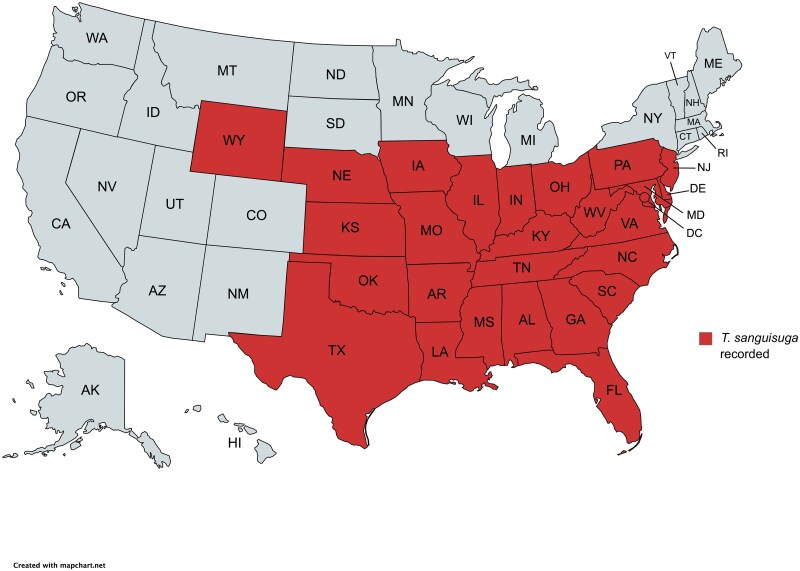
Geographical distribution of *T. sanguisuga.* Map created with mapchart.net.

The most widespread triatomine species in the United States is *Triatoma sanguisuga* (Fig. 1). The species has been recorded in 26 states from the southernmost states up to about the 42nd parallel, from the Rocky Mountains to the eastern seaboard (Triatoma sanguisuga in Iowa 2017; Centers for Disease Control and Prevention 2019; Reeves and Miller 2020; Nielsen *et al*. 2021) (Fig. 2). Spanning over 1 million square miles, *T. sanguisuga* is found in different ecoregions including most of the Great Plains, the eastern temperate forests, and the tropical wet forests of southern Florida (United States Environmental Protection Agency 2024).

*T. sanguisuga* is considered an epidemiologically relevant disease vector species in the United States because it can be found in domestic and peridomestic habitats and it has been implicated in autochthonous (i.e. vector-borne) Chagas disease cases (Dorn *et al*. 2007; Beatty and Klotz 2020; Lynn *et al*. 2020). Prior scientific studies of *T. sanguisuga* took place mainly in 3 of the 24 states in which the species is found: Louisiana, Texas, and Florida. There, *Try. cruzi* infection prevalences upwards of 60% have been found in *T. sanguisuga* (Cesa *et al*. 2011; Moudy *et al*. 2014; Curtis-Robles *et al*. 2018), and blood meal analyses have revealed that the species feeds upon a wide range of taxa comprising reptiles, birds, amphibians, and mammals, including humans (Waleckx *et al*. 2014; Dumonteil *et al*. 2018, 2020, 2024; Balasubramanian *et al*. 2022).

Despite its ubiquity and epidemiologic importance, little is known about the genetic variation in *T. sanguisuga;* individuals are assigned to the species solely based on morphology and geographic location (De Paiva *et al*. 2022). Five *T. sanguisuga* subspecies have been suggested based on morphological variation, and 2 of these subspecies were eventually assigned to a new species, *T. indictiva* Neiva 1912. The 3 remaining subspecies were eventually rejected, but uncertainty still exists (De Paiva *et al*. 2022). Two small studies of *T. sanguisuga* found a high level of genetic diversity between populations. A comparison of cytochrome oxidase II mitochondrial gene sequences from 33 *T. sanguisuga* specimens collected in 2 barrier islands off the southern coast of Georgia revealed 12 distinct haplotypes with no haplotypes shared between populations from different islands, suggesting limited dispersal and genetic exchange (Roden *et al*. 2011). De La Rua *et al*. (2011) investigated intraspecific genetic variation and population structure in 54 *T. sanguisuga* specimens collected in rural New Orleans, LA, USA, and found 2 groups that were genetically divergent enough to represent different subspecies. The high degree of genetic variation discovered in these 2 studies hints at a wealth of diversity waiting to be discovered in *T. sanguisuga* considering that its geographic range spans multiple ecological regions with varying seasonality, habitat, and other environmental drivers of genetic diversity.

Here, we present the *T. sanguisuga* genome, which is the first genome sequenced for any endemic North American triatomine species. Our whole-genome sequencing of *T. sanguisuga* will facilitate comparative analyses between populations to resolve questions of species. In addition, insights into the genetic underpinnings of vector behavior and physiology can lead to new vector control targets. Thus, the *T. sanguisuga* genome will contribute to genetic studies of epidemiologically relevant characteristics of *T. sanguisuga* such as blood feeding, host seeking, parasite competence, and domiciliation (Abad-Franch and Monteiro 2005; Fitzpatrick *et al*. 2008; Mesquita *et al*. 2015) and in turn increase our public health preparedness.

## Materials and methods

### Specimen origin

The *T. sanguisuga* specimen used in this study was 1 of 2 adult specimens (a male and a female) captured within a home in New Castle County, DE, USA, as detailed in the study by Peterson *et al*. (2024). The specimens were given to our lab at the University of Delaware by the homeowner. The species was confirmed geographically (*T. sanguisuga* is the only species recorded for the northern United States) and morphologically using the key in (Carcavallo *et al*. 1998), which distinguishes *T. sanguisuga* by the length of its second rostral segment, the coloring of the posterior lobe of the pronotum and humeri and the insects’ size. The intestinal contents of both specimens were tested for *Try. cruzi* infection via real-time PCR as described in the studies by Muñoz-Calderón *et al*. (2020) and Peterson *et al*. (2024). The male specimen tested positive for *Try. cruzi* infection and the female specimen tested negative. The legs and head of the *T. sanguisuga* specimen that tested negative for *Try. cruzi*, which was the female specimen, were used for sequencing the genome presented in this study.

### DNA extraction and sequencing

High molecular weight (HMW) genomic DNA extraction and purification from the sample was performed using the MagAttract HMW DNA Kit (Qiagen Inc., Venlo, Netherlands) as per manufacturer’s instructions. HMW DNA was confirmed using a FemtoPulse (Advanced Analytical Technologies Inc., Ankeny, IA, USA). The HMW DNA (10 μg aliquots) were converted to SMRTbell templates using the SMRTbell prep kit 3.0 (Pacific Biosciences, Menlo Park, CA, USA) as per manufacturer’s instructions. Briefly, samples were end-repaired and ligated to blunt adaptors. Exonuclease treatment was performed to remove unligated adapters and damaged DNA fragments. Samples were purified using 0.6× AMPureXP beads (Beckman Coulter Inc., Brea, CA, USA). The purified SMRTbell libraries were eluted in 10 μL of elution buffer. Eluted SMRTbell libraries were size selected on the BluePippin (Sage Science Inc., Beverly, MA, USA) to eliminate library fragments below ∼10 kb. Final library quantification and sizing was carried out on a FemtoPulse (Advanced Analytical Technologies Inc.) using 1 μl of library. The amount of primer and polymerase required for the binding reaction was determined using the SMRTbell concentration and library insert size. Primers were annealed, and polymerase was bound to the SMRTbell template. Sequencing was performed using the Revio platform (Pacific Biosciences). The HiFi libraries were run on Revio system 25M SMRT cells using sequencing chemistry 3.0 with 4-h preextension and 30-h movie time.

### Comparative analysis

Whole-genome sequences have been assembled for 4 other species in the subfamily Triatominae: *Rhodnius prolixus* (NCBI Bioproject ID PRJNA13648), *T. infestans* (NCBI Bioproject ID PRJNA589079), *T. dimidata* (NCBI Bioproject ID PRJEB77418), and *T. rubrofasciata* (NCBI Bioproject ID PRJNA516044). Although now eliminated from the region, *R. prolixus* is believed responsible for the majority of current Chagas disease cases in Central America (Hashimoto and Schofield 2012; Peterson, Hashimoto, *et al*. 2019; Peterson, Yoshioka, *et al*. 2019), and it is still one of the main vectors of Chagas disease in Colombia and Venezuela (Fitzpatrick *et al*. 2008; Méndez-Cardona *et al*. 2022). *T. infestans* is found in the southern cone of South America, and it is one of the main vectors of Chagas disease cases in that region (Coura 2015). Note that 2 additional genomes of *T. infestans* have reportedly been sequenced (Pita *et al*. 2017; Pita *et al*. 2017; Pita *et al*. 2018; Mora *et al*. 2023), but the data are not publicly available. Thus for quantitative comparison, we used data from the publicly available *T. infestans* genome sequence. *T. dimidiata* is arguably the most epidemiologically important Triatominae species in Central America, where it is the main vector responsible for vector-borne Chagas disease cases since the elimination of *R. prolixus* (Peterson, Hashimoto, *et al*. 2019).

The 4th species in our comparative analyses, *T. rubrofasciata*, is classified as a congeneric species to *T. sanguisuga*, but it has a much wider geographic distribution that spans both the old world and the new world, while *T. sanguisuga, T. infestans*, and *R. prolixus* are limited to just the new world. The origin of *T. rubrofasciata* is disputed, with some researchers arguing for a new world origin (Gorla *et al*. 1997) and others, an old world origin (Ryckman and Archbold 1981). The species is associated with ship rats, and the mechanism underlying its global dispersal from its place of origin is hypothesized to be ancient maritime trade, as it is found in port cities throughout Asia and Africa. There, *T. rubrofasciata* is a nuisance species, invading homes and causing painful and sometimes allergenic bites (Dujardin *et al*. 2015). However, *T. rubrofasciata* is not known to spread *Try. cruzi* outside of the Americas and is therefore not of high epidemiological interest as a Chagas disease vector in the old world. The *T. rubrofasciata* specimen from which the genome was sequenced was collected in Foshan City in the Guangdong Province of China (Liu *et al*. 2019). In this study, *T. rubrofasciata* is the most geographically distant of the 4 comparison Triatominae species, and it is the only specimen originating in the old world.

### Genome assembly methods

Quality control of the HiFi PacBio reads was performed using NanoPlot v1.43.0 (De Coster and Rademakers 2023). Due to the high quality of the reads, no filtering was needed. The de novo assembly of the *T. sanguisuga* genome was performed using Flye v2.9 (Kolmogorov *et al*. 2019), Hifiasm v0.19.5 (Cheng *et al*. 2021), and PacBio’s assembly pipeline in SMRT Link portal v13.1.0 (https://www.pacb.com/smrt-link/). The Quality Assessment Tool (QUAST) v5.1.0 (Mikheenko *et al*. 2018) and the Benchmarking Universal Single-Copy Orthologs (BUSCO) software v5.4.7 (Manni *et al*. 2021) were used to assess the completeness of each assembly and compare them with the genome assemblies of *R. prolixus* (GCA_000181055.3), *T. infestans* (GCA_011037195.1), *T. dimidata* (GCA_964198125), and *T. rubrofasciata* (data available in the study by Liu *et al*. 2019). Briefly, QUAST calculates and compares genome assembly quality metrics, with or without a reference sequence. The BUSCO software examines genomic assembly quality and completeness based on expected single-copy orthologs. BUSCO was run using the “hemiptera_odb10” lineage, which consisted of 2,510 single-copy orthologs. The best assembly (the Hifiasm primary assembly) was selected for repeat masking and gene annotation. Assembly polishing was carried out with POLCA genome polishing software (Zimin and Salzberg 2020), which is part of the The Maryland Super Read Cabog Assembler (MaSuRCA) genome assembly and analysis toolkit v4.1.0 (Zimin *et al*. 2013). Full commands for all bioinformatics steps are provided in Supplementary File 1.

### Decontamination

The Basic Local Alignment Search Tool (BLASTn v2.15.0+; Altschul *et al*. 1990; Camacho *et al*. 2009) was used to query the contigs against the BLAST core_nt database v1.1 to check for contamination. Briefly, the core_nt database is a large, publicly available nucleotide sequence database managed by the National Center for Biotechnology Information (NCBI). Hits with an *e*-value cutoff of 0.01 and longer than 200 bp were examined manually. We set lenient thresholds for both the *e*-value and size so as not to miss anything during manual review. Contigs with only hits to insects and ribosomal RNA were not considered contamination. All other contigs represented possible contamination and were examined more closely via online BLASTn, taking into account contig coverage, alignment length, and percent identity.

### Mitochondria identification

First, BLAST was used to query the contigs against the existing *T. sanguisuga* mitochondrial sequence (NC_050329.1). Hits were filtered by query coverage to retain contigs with more than 50% of their sequence aligning to the mitochondrial sequence. To confirm the possible mitochondrial contigs and select a representative, MitoHifi v3.2.2 (Uliano-Silva *et al*. 2023) was run via its Singularity container. MitoHifi was also used to annotate and circularize the representative contig and modify it to start at transfer RNA (tRNA)-Phe. The BLAST alignment between the representative contig and the existing *T. sanguisuga* mitochondrial sequence was used to identify any regions in the representative contig that was not in the reference mitochondrial sequence. Identified significant region(s) were extracted using bedtools and queried against nt and Univec build 10 (https://www.ncbi.nlm.nih.gov/tools/vecscreen/univec/) databases to determine the potential source of the sequence. Any region with no clear source (no significant hits to either database) was removed. The resulting sequence was reannotated with MitoHifi and visualized using circularMT (Goodman and Carr 2024).

### MtDNA phylogenetic analysis

As with mitochrondria identification, the first step of our mtDNA comparison was a BLAST search using our *T. sanguisuga* mtDNA genome sequence. We then downloaded the full mtDNA genome sequences of the closest 9 named species (all in the genus *Triatoma*) yielded by the BLAST search. We aligned all sequences and built a phylogeny with RAxML v.8.2.11 (Stamatakis 2014) in Geneious v.11.1.5 (https://www.geneious.com/). A best scoring maximum likelihood phylogeny was generated with support values based on 100 bootstrap replicates. The phylogeny was midpoint rooted using the function midpoint() in the R package phangorn v.2.11.1 (Schliep 2011) and plotted with functions in the R package ape v.5.7-1 (Paradis and Schliep 2019).

### Methylation

PacBio sequencing enables detection of 5-methylated cytosines (5mCs; Flusberg *et al*. 2010). Hifi reads with the 5mC information (5mC tags in an unaligned bam file) were aligned to the Hifiasm primary assembly using Pbmm2 v1.14.0 (https://github.com/PacificBiosciences/pbmm2), a C++ wrapper for minimap2 v2.26 (Li 2018). PacBio’s pb-CpG-tools v2.3.2 (https://github.com/PacificBiosciences/pb-CpG-tools) was then used to produce per-site methylation probabilities from the alignment. Global CpG methylation was calculated by dividing the number of methylated CpG cytosines by the number of unmethylated cytosines in the reads that aligned to the genome assembly.

### Repeat assembly techniques

Repeats in the Hifiasm primary assembly were identified and modeled using RepeatModeler v2.0.3 (Flynn *et al*. 2020). Repeats from this de novo identification were masked using RepeatMasker v4.1.2 (Smit *et al*. 2013). In addition, RepeatMasker was run using Dfam database v3.2 and the parameter “-species Triatoma.” Complex repeats were extracted from the RepeatMasker results and given to the Maker annotation pipeline (Cantarel *et al*. 2008; Campbell *et al*. 2014) for hard-masking (see next section).

### Gene finding methods

Annotation was performed using Maker v3.01.04 (Cantarel *et al*. 2008). First, an evidence-based round of Maker was run using the transcripts from *R. prolixus* obtained from VectorBase release 68 (Giraldo-Calderón *et al*. 2015) and the “Triatominae” canonical proteins from UniProt release 2024_3 (Apweiler *et al*. 2004; The UniProt Consortium 2023) as the transcript and protein evidence, respectively. For the repeat masking parameters of Maker, the “rm_gff” parameter was set to the complex repeats GFF from RepeatMasker, “model_org” was set to “simple,” and “repeat_protein” was set to Maker’s provided “te_proteins.fasta” file. The transcripts (with added 1,000 bp flanking regions) from this first round of Maker were then used to train gene models using Augustus v3.5.0 (Stanke *et al*. 2006) via BUSCO v5.4.7 long mode. The starting Augustus species was set to “rhodnius.” The new Augustus model was then provided into a second round of Maker to produce the final set of predicted gene annotations. Putative gene function was assigned by following Maker Support Protocols 2 and 3 (Campbell *et al*. 2014), and functional domains and gene ontology (GO) terms were assigned via Interproscan v5.53-87.0 (Blum *et al*. 2021) by following steps 4 and 5 of Basic Protocol 5 (Campbell *et al*. 2014). The predicted genes were then sorted into a high confidence set by filtering out genes with no function assigned and an annotation edit distance of 1 (no transcript or protein evidence from Maker). BUSCO with the “hemiptera_odb10” lineage was used to assess the completeness of the high confidence proteins and transcripts. The quality of the annotated proteins was also examined by identifying their *R. prolixus* and *T. rubrofasciata* orthologs using the OrthoVenn3 web service (Sun *et al*. 2023) with the Orthofinder algorithm (Emms and Kelly 2019). OrthoVenn3 was used to retrieve significantly (*P* < 0.05) enriched GO terms for the ortholog clusters unique to *T. sanguisuga*. All annotation files are located in Supplementary File 2.

## Results and discussion

### Assembly

A total of 91.08 Gb of sequence was generated with an N50 of 14,271.0 bp. Ninety-two percent of reads were above the Q20 quality cutoff, and 74.6% were above the Q25 cutoff (Table 1). The Hifiasm assembly was selected for annotation based on a comparison of quality statistics and BUSCO scores among the Flye, Hifiasm, and Smrtlink assemblies, as described above (Table 2). The best assembly was the Hifiasm assembly, which was 1.165 Gb spread over 282 contigs. Decontamination revealed 2 contigs not pertaining to *T. sanguisuga*; the 1st contig was aligned (>90% identity) with sequences from mitochondria of 3 fungal species belonging to the order Hypocreales, which includes several entomopathogenic species. The 2nd contig was aligned to a tick-borne protozoan species, *Hepatozoon canis*. Both contigs were removed from the Hifiasm assembly, which decreased the genome size by 105,876 bp (Table 2). Analysis of the mitogenome revealed 98 redundant contigs, 97 of which were removed, resulting in a final assembly of 1.162 Gb spread over 183 contigs with an N50 of 94,972,618 bp and coverage of 77.7×. Repeat analysis using the RepeatModeler and RepeatMasker software revealed that the *T. sanguisuga* genome consists of 62.75% repetitive DNA, with 41.6240.58% interspersed repeats, 2.345% simple repeats, and 0.46% low complexity repeats (Table 3). The genome-wide GC level was 33.56%. All sequence and assembly data, including sequences of the 2 contaminated contigs that were removed, are available under NCBI BioProject ID PRJNA1140168 and accession number SRR29988702.

**Table 1.** Quality cutoff data for *T. sanguisuga* sequencing reads.

Quality cutoff	# of reads above cutoff	% of reads above cutoff	Mb of reads above cutoff
Q10	6,532,647	100.0	91,075.0
Q15	6,529,241	99.9	91,027.9
Q20	6,021,510	92.2	83,537.3
Q25	4,872,187	74.6%	66,254.9
Q30	3,438,652	52.6	44,709.0

**Table 2.** Quality statistics for assemblies of the *T. sanguisuga* genome by 3 different de novo assemblers.

	Flye	Smrtlink	Hifiasm (draft 1)	Hifiasm decontaminated (draft 2)	Final assembly
Assembly					
Length (bp)	2,079,745,197	1,180,738,419	1,165,190,363	1,165,084,487	1,162,099,166
Contigs	9,026	1,068	282	280	183
Largest contig	7,311,449	24,892,402	144,379,499	144,379,499	144,379,499
Min contig length	1,021	1,811	9,031	9,031	9,031
Mean contig length	230,417.2	1,105,560.3	4,131,880.7	4,161,016	6,350,268.7
N50	388,970	6,977,916	94,972,618	94,972,618	94,972,618
L50	1,563	52	5	5	5
BUSCO					
Complete	98.9%	98.8%	99.1%	99.1%	99.1%
Single	36.4%	97.8%	97.8%	97.8%	97.8%
Duplicate	62.5%	1.1%	1.3%	1.3%	1.3%
Fragment	0.7%	0.7%	0.6%	0.6%	0.6%
Missing	0.4%	0.4%	0.3%	0.3%	0.3%
*n*	2,510	2,510	2,510	2,510	2,510

The Hifiasm draft 1 assembly was selected as the best assembly and thereafter underwent decontamination to produce the draft 2 assembly, followed by removal of 97 redundant contigs pertaining to the *T. sanguisuga* mitochondria to produce the final assembly.

**Table 3.** Repetitive DNA in the *T. sanguisuga* genome.

*Name*	Number of elements^a^	Length	Percent of genome
Retroelements	869,571	229,854,099 bp	**19**.**78**
SINEs	66,050	6,690,116 bp	0.58
Penelope class	88,144	15,173,646 bp	1.31
LINE class	774,151	193,638,471 bp	16.66
CRE/SLACS	0	0 bp	0.00
L2/CR1/Rex	34,854	20,442,282 bp	1.76
R1/LOA/Jockey	390,559	77,649,217 bp	6.68
R2/R4/NeSL	8,077	3,140,410 bp	0.27
RTE-Bov-B	100,694	35,959,107 bp	3.09
L1/CIN4	91	49,474 bp	0.00
LTR elements	29,370	14,351,866 bp	1.23
BEL/Pao	6,426	2,789,295 bp	0.24
Ty1/Copia	8,706	1,639,481 bp	0.14
Gypsy/DIRS1	14,145	9,880,325 bp	0.85
Retroviral	0	0 bp	0.00
DNA transposons	509,635	84,165,895 bp	**7**.**24**
hobo-Activator	50,682	15,582,243 bp	1.34
Tc1-IS630-Pogo	154,823	27,948,296 bp	2.40
En-Spm	0	0 bp	0.00
MuDR-IS905	0	0 bp	0.00
PiggyBac	3,233	1,198,104 bp	0.10
Tourist/harbinger	2,099	674,150 bp	0.06
Other (mirage, P-element, transib)	189	86,932 bp	0.01%
Unclassified	649,059	169,616,531 bp	**14**.**60**
Total interspersed repeats		483,636,525 bp	41.62
Rolling-circles	1,198,797	209,049,142 bp	17.99
Small RNA	24,593	4,690,086 bp	0.40
Satellites	7,833	1,134,749 bp	0.10
Simple repeats	693,752	27,246,855 bp	2.34
Low complexity	107,640	5,319,819 bp	0.46
Total repetitive DNA, % of bp analyzed (1.162 Gb)		731,007,176	62.91
Total repetitive DNA, % of whole genome (1.165 Gb)		731,007,176	62.75

Sequences analyzed: 183, comprising 99.7% of bp (1.162 Gb).

^a^Most repeats fragmented by insertions or deletions have been counted as 1 element. Total interspersed repeats are calculated by adding the numbers shown in bold. All percentages except the total were calculated as a percent of all base pairs analyzed (1.162 Gb). The total is shown as a percent of all bp analyzed and % of the total genome.

### Quality, completeness, and coverage

**Fig. 3.**
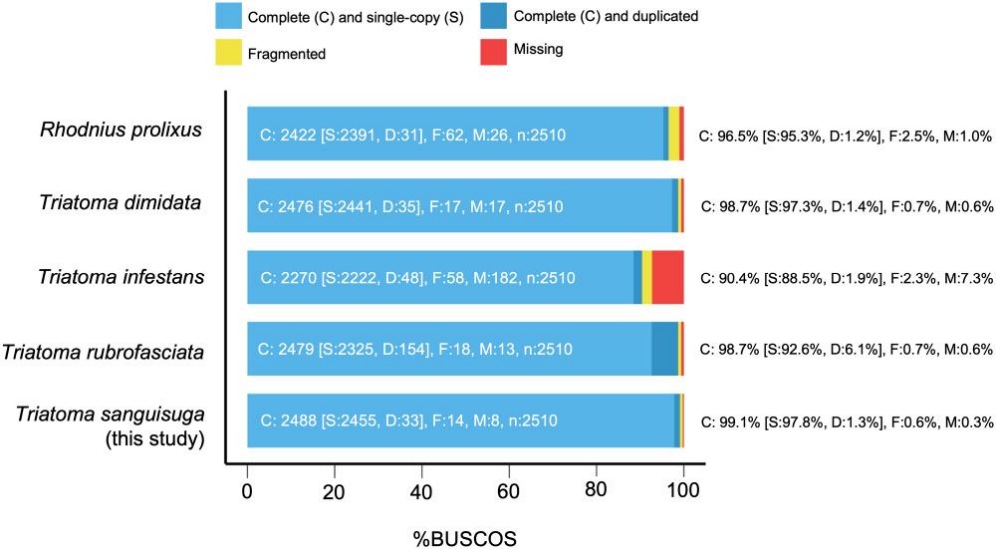
Genome BUSCO scores for assembled *Triatominae* genomes. *T. sanguisuga* shown is from this study.

Keeping in mind that accuracy of an assembly without an existing reference for a species is difficult to assess, our assembly appears to be high quality. The consensus quality reported by the POLCA software before any polishing was 99.9996%; subsequent polishing through POLCA did not improve the quality, so we moved forward with the nonpolished assembly. At 1.162  Gb with no gaps, the *T. sanguisuga* genome is very similar in size to that of *T. dimidiata*, which was measured to be 1.220 Gb, suggesting a high level of completeness. The *T. sanguisuga* genome size is slightly larger than the other 3 comparison species (*R. prolixus*: 706.8 Mb, *T. infestans*: 949 Mb, and *T. rubrofasciata*: 681 Mb; Table 4), but still falls within an expected range. The *R. prolixus* genome was the first triatomine species genome ever assembled, in 2015. Given advances in the field during the past 10 years, the *R. prolixus* genome published had over 142 million unknown bases (Ns), while our genome did not have any unknown bases, which might explain some of the size difference. The GC percentage was similar between all species, varying by less than half a percentage point. With 183 contigs, our assembly is the more contiguous than that of *T. infestans* and *R. prolixus*, with an N50 that is roughly 87 times larger than *R. prolixus* and 873 times larger than *T. infestans* (Table 4). We have fewer contigs than the assemblies for *T. dimidiata* and *T. rubrofasciata*, but they each include 13 chromosome scaffolds, while the contigs of our assembly do not have chromosome assignments. At 99.1%, our BUSCO score indicates excellent quality in terms of completeness that is slightly higher than that of *T. dimidiata* and *T. rubrofasciata*, both 98.7%, and higher than *R. prolixus* and *T. infestans*, which were 96.5 and 90.4%, respectively (Fig. 3).

**Table 4.** Comparison of quantitative genome characteristics among *T. sanguisuga* (from this study), *T. dimidiata*, *T. infestans*, *R. prolixus*, and *T. rubrofasciata*.

	*T. sanguisuga*	*T. dimidiata*	*T. infestans*	*R. prolixus*	*T. rubrofasciata*
Summary statistics					
Contigs	183	1,098 (includes 13 chromosome scaffolds)	14,951	16,511	1,174 (includes 13 chromosome scaffolds)
Contigs >5,000 bp	183	1,089	14,849	4,598	1,001
Contigs >10,000 bp	182	1,043	13,959	3,097	793
Contigs >25,000 bp	151	680	9,815	1,862	418
Contigs >50,000 bp	48	396	5,919	1,089	158
Largest contig	144,379,499 bp	147,806,421 bp	1,054,224 bp	13,425,595 bp	97,329,580 bp
Total Length	1,162 Mb	1,220 Mb	949 Mb	707 Mb	681 Mb
N50	94,972,618	92,900,019	108,830	1,088,772	50,700,875
GCs %	33.56	33.96	34.03	33.94	33.39
Coverage	77.7×	48.0×	200.0×	8.3×	68.6× (short read), 101.9× (long read)
#Ns per 100 kb	0	8.62	342.49	20,118	60.45
#Ns	0	104,200	3,251,881	142,194,158	411,500

Metrics (except for total length) are calculated after filtering out contigs <1,000 bp long.

### Genome annotation

**Fig. 4.**
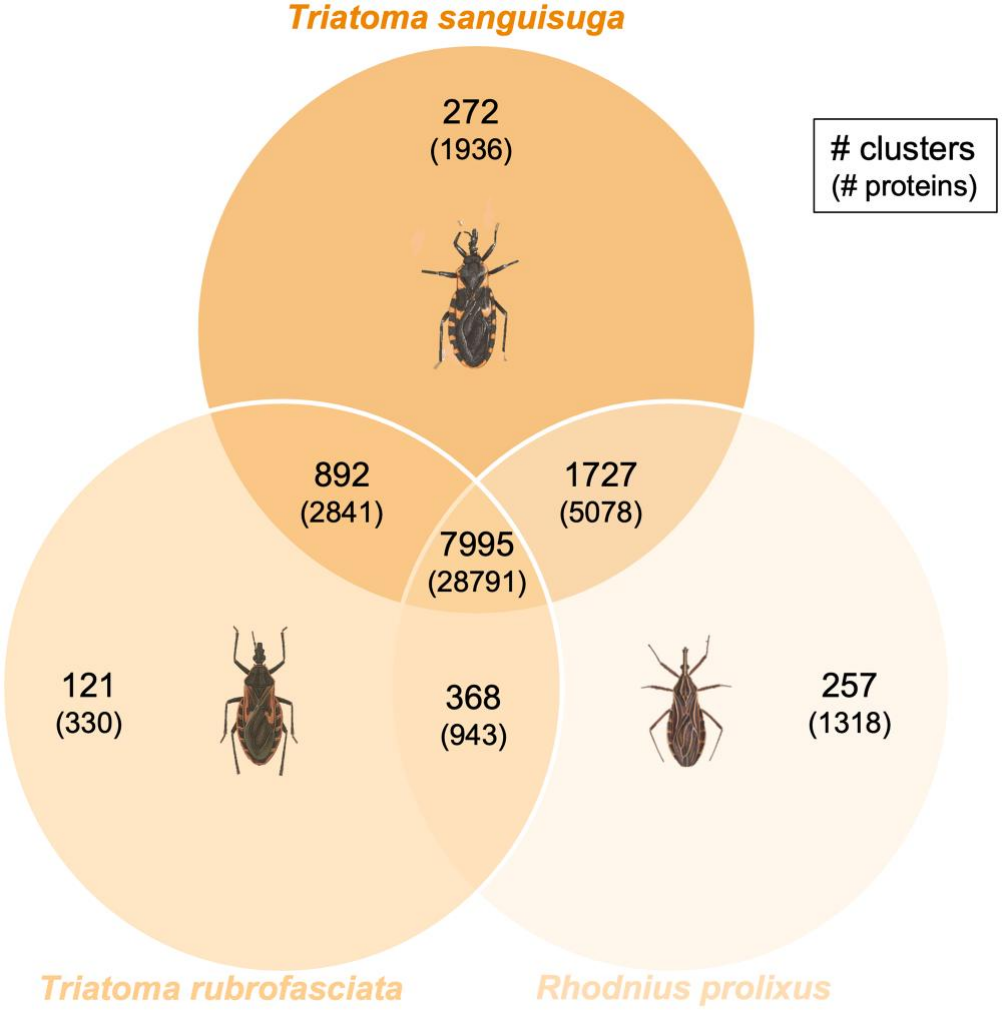
Protein count and cluster count overlaps for *T. sanguisuga*, *R. prolixus*, and *T. rubrofasciata*. Drawing of *T. sanguisuga* by Laura Ulrich (used with Ms. Ulrich’s permission.); *T. rubrofasciata* image drawn by Brazilian scientific artist Castro Silva (ca. 1930s). *R. prolixus* image: Centers for Disease Prevention and Control Southeastern Center of Excellence in Vector-Borne Diseases.

We detected 17,799 putative protein-coding genes using the Maker genome annotation pipeline (Table 5). The final protein BUSCO score was 94.4% complete (92.4% single copy and 2.0% duplicates). This number is comparable with that of *R. prolixus*, which was predicted to have 15,456 protein-coding genes. In a comparative analysis of *T. sanguisuga* proteins with *R. prolixus* reference proteins and *T. rubrofasciata* reference proteins using the web service OrthoVenn3 (Sun *et al*. 2023), 7,995 orthologous protein clusters comprising 28,791 proteins were found to overlap among all 3 species (Fig. 4). Interestingly, *T. sanguisuga* shared more protein clusters with *R. prolixus* than with its congeneric *T. rubrofasciata*. Between just *T. sanguisuga* and *T. rubrofasciata*, 892 overlapping protein clusters comprising 2,841 proteins were found, while 1,727 protein clusters comprising 5,078 proteins were shared between T. sanguisuga and *R. prolixus*. It is possible that the differences could be attributed to factors relating to differences in geographic location (China for *T. rubrofasciata* vs the Americas for *T. sanguisuga* and *R. prolixus*), but further molecular comparisons with these species are needed to identify genomic differences between new world and old world *Triatoma* species.

**Table 5.** Quantitative summary of the annotated final genome of *T. sanguisuga*.

Feature	Count
Genes	17,799
Transcripts/proteins	17,799
Exons	102,331
Introns	84,532
Mean gene length	8,010
Mean exons per transcript	5.7
Single exon transcripts	3,348
Proteins with predicted function	13,696
Lower confidence proteins	9,723

### Unique gene families and functions

**Fig. 5.**
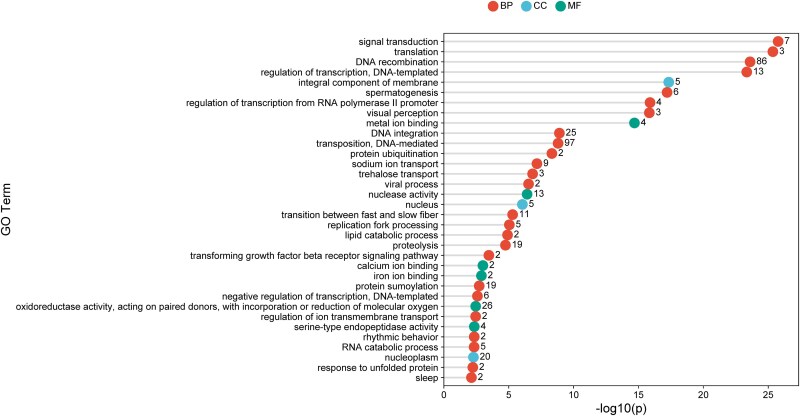
Lollipop chart showing significantly (*P* < 0.05) enriched GO terms for the ortholog clusters unique to *T. sanguisuga*. *X* axis: the logarithmic scale of the adjusted *P*-values in enrichment GO analysis. (Here, the *P*-value is the probability of seeing at least multiple number of genes out of the total *n* genes in the unique clusters annotated with a particular GO term, given the proportion of genes in the whole genome that are annotated with that GO term.) *Y* axis: GO terms. The number of proteins in the cluster(s) associated with each GO term are shown next to each circle. Abbreviations represent the three GO categories: biological processes (BP), cellular components (CC), and molecular functions (MF). Plot is created using SRplot software (Tang *et al*. 2023).

Preliminary analysis of enriched GO terms for the ortholog clusters unique to *T. sanguisuga* revealed 34 significantly enriched GO functional categories comprised of 418 proteins (Fig. 5). The most significant GO category was signal transduction (*P* < 1*e*−25), which comprised 7 proteins. The majority of functional categories pertained to biological processes and the most abundant proteins corresponded to DNA mediated transposition functions (97 proteins) and DNA recombination (86 proteins) functions. Functions of potential epidemiological interest found in the analysis include those possibly related to hematophagy (e.g. iron ion binding, lipid catabolic process), host seeking and foraging (e.g. visual perception, clock functions, rhythmic behavior), and reproduction (e.g. spermatogenesis). Further comparative genomic analyses between these species could also reveal important differences that have allowed *T. sanguisuga* to withstand the cooler winters found in northern North America when compared with the more tropical environments to which *R. prolixus* and *T. rubrofasciata* have evolved.

### Mitochondrial structure

**Fig. 6.**
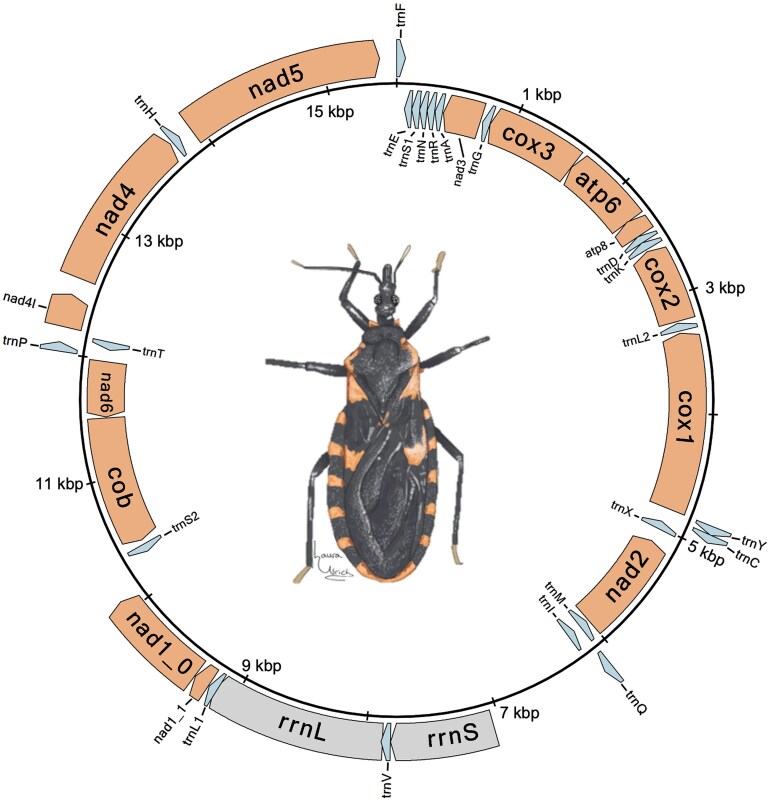
Annotated mitogenome for *T. sanguisuga*. Protein coding genes are shown in orange, tRNA genes are shown in light blue, and rRNA genes are shown in gray. Mitogenome was drawn using circularMT (Goodman and Carr 2024). Drawing of *T. sanguisuga* by Laura Ulrich (used with Ms. Ulrich’s permission).

Ninety-eight contigs from the original Hifiasm assembly were identified as redundant copies of the *T. sanguisuga* mitogenome. The representative mitochondrial contig selected and circularized by MitoHifi was aligned to the existing *T. sanguisuga* mitochondrial genome (NC_050329.1). BLAST hits between the representative contig and the existing *T. sanguisuga* mitochondrial sequence showed that there was an additional ∼1,500 bp in the representative contig. This ∼1,500 bp was extracted and queried against nt and Univec build 10 databases. With no significant hits to either database, the region was subsequently removed from the contig. The final annotated mitogenome assembly was a single circular contig measuring 15,542 bp in length with 14 protein-coding genes, 2 ribosomal RNAs (rRNAs), and 22 tRNAs (Fig. 6). The sequence matched the reference sequence with 95.49% identity over 99% of its length.

### Mitochondrial comparison

**Fig. 7.**
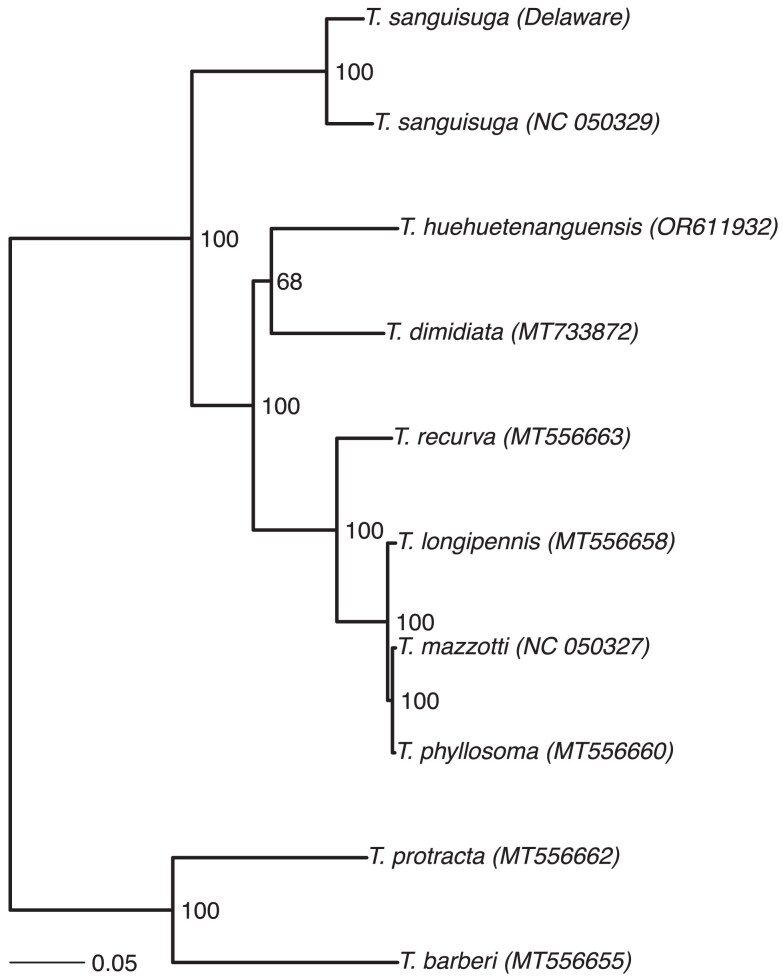
Phylogenetic relationships among full mitochondrial Triatominae genomes with the *T. sanguisuga* from this study and 9 close relatives. Node values are bootstrap support percentages. The units shown on the scale bar are nucleotide substitutions per site. The GenBank accession number of each genome is listed in parentheses after each species names; the sample from this study has “Delaware” listed after the species name.

A phylogenetic analysis of full mitochondrial genomes demonstrated that the *T. sanguisuga* specimen from this study is most closely related to another *T. sanguisuga* specimen in GenBank (Fig. 7). Interestingly, several named Triatominae species in our analysis were more closely related in their mitochondrial genomes (*T. mazzotti, T. phyllosoma, T. longipennis*; Fig. 7) than the 2 *T. sanguisuga* specimens. This finding suggests that the 2 *T. sanguisuga* specimens may represent cryptic species, or perhaps a species with highly diverged populations, highlighting the importance of reassessing species limits with molecular methods. Further analyses (phenotypic and genomic) are needed to resolve taxonomic relationships between specimens denoted as *T. sanguisuga*.

### Conclusion

Here, we present the first genome sequence for *T. sanguisuga*, which is also the first whole-genome sequence for any US or North American triatomine species. The *T. sanguisuga* genome will facilitate the identification of genetic differences between *T. sanguisuga* populations, plausibly with relevance to epidemiologically important traits. Vector reference genomes contribute to our ability to carry out genetic investigations of physiological and behavioral attributes of disease vectors such as blood feeding, host seeking, and parasite competence. Findings from such studies can be used to guide vector-borne disease management strategies and in turn strengthen public health preparedness.

## Supplementary Material

jkae308_Supplementary_Data

## Data Availability

Supplementary File 1, submitted with this manuscript, contains the full commands for all bioinformatics steps. Supplementary File 2 is available on GSA FigShare (https://doi.org/10.25387/g3.26939842) and contains the annotation files. Sequence data and the genome assembly are publicly available on NCBI under BioProject ID PRJNA1140168 and accession number SRR2998870. Supplemental material available at G3 online.
